# The Concomitant Effect of the Antiepileptic Drug Lacosamide and rTMS on an SH-SY5Y Model of Neuronal Excitability

**DOI:** 10.3390/neurolint17100152

**Published:** 2025-09-24

**Authors:** Ioannis Dardalas, Efstratios K. Kosmidis, Vasilios K. Kimiskidis, Roza Lagoudaki, Theodoros Samaras, Theodoros Moysiadis, Dimitrios Kouvelas, Chryssa Pourzitaki

**Affiliations:** 1 Laboratory of Clinical Pharmacology, Aristotle University of Thessaloniki, 54124 Thessaloniki, Greece; 2 Laboratory of Physiology, Department of Medicine, Aristotle University of Thessaloniki, 54124 Thessaloniki, Greece; 3 First Department of Neurology, AHEPA University Hospital, Aristotle University of Thessaloniki, Stilponos Kyriakidi 1, 54636 Thessaloniki, Greece; 4 Faculty of Sciences, School of Physics, Aristotle University, 54124 Thessaloniki, Greece; 5 Department of Physics, University of Malta, MSD 2080 Msida, Malta; 6 Department of Computer Science, School of Sciences and Engineering, University of Nicosia, 2417 Nicosia, Cyprus

**Keywords:** rTMS, lacosamide, epilepsy, antiepileptic drug, in vitro, cell culture, calcium dysregulation

## Abstract

**Background/Objectives**: Epilepsy is identified by irregular neuronal hyperexcitability, generating recurrent seizures. Despite many available pharmacological treatments, certain patients with drug-resistant epilepsy may require novel therapeutic approaches. In the present study, we aimed to evaluate the effects of lacosamide, low-frequency repetitive transcranial magnetic stimulation, and their combination on intracellular calcium dynamics in an in vitro model of neuronal excitability, hypothesizing that these interventions could mitigate potassium chloride-induced neuronal excitation. **Methods**: We utilized differentiated SH-SY5Y human neuroblastoma cells as an in vitro model of neuronal excitability. Neuronal excitability was induced with 50 mM KCl, and cells were treated with lacosamide (300 µM), rTMS (1 Hz), or their combination. Intracellular calcium levels were quantified using fluo-4 AM fluorescence calcium imaging, with changes expressed as percentage change in fluorescence intensity (%ΔF/F) relative to baseline. **Results**: The combination of lacosamide and rTMS was the most effective, significantly reducing KCl-induced calcium elevation (ΔF/F = 9.15) compared to lacosamide alone (ΔF/F = 17.11), rTMS alone (ΔF/F = 23.70), and the untreated cells serving as controls (ΔF/F = 66.70). The combination showed a statistically significant effect, with enhanced suppression of neuronal excitability compared to individual treatments. **Conclusions**: Lacosamide and low-frequency rTMS (1 Hz) effectively attenuated KCl-induced changes in intracellular calcium levels in vitro, with their combination demonstrating the highest efficacy. These findings suggest a promising foundation in the management of drug-resistant epilepsy. Future studies are necessitated to validate these results and benefit clinical translation.

## 1. Introduction

Over 100 million people around the globe are affected by the neurological disorder known as epilepsy. About one-fifth to one-third of the patients with epilepsy, using a monotherapy or a combination of antiepileptic medicines, will not be adequately treated for their condition. Some of them will remain resistant to any given pharmacological treatment, unable to control the related seizures, having a low quality of life [1].

Novel therapeutic approaches have emerged in recent decades, including new antiepileptic drugs with different mechanisms of action, offering a favorable profile in terms of efficacy, tolerability, and safety, with few side effects [2]. Early antiepileptic drugs include phenobarbital, phenytoin, valproate, carbamazepine, while new generation antiepileptic drugs include pregabalin, lamotrigine, levetiracetam, lacosamide, brivaracetam, and topiramate [2]. Non-pharmacological approaches, already used in clinical practice, involve the use of magnetic resonance imaging (MRI)-guided laser thermal ablation and repetitive transcranial magnetic stimulation (rTMS). These non-pharmacological approaches may have a significant impact on the management and treatment of drug-resistant epilepsy (DRE) [3,4].

Epileptogenesis is the process by which spontaneous, recurrent seizures develop in a normal neuronal network, influenced by multiple factors that may disturb neuronal homeostasis and stimulate hyperexcitability. Intracellular signaling transduction is linked to epileptogenesis. CREB, a cAMP-dependent transcription factor, is a vital neuronal protein that facilitates long-term memory and cell survival. It is activated by distinct signaling pathways involving cAMP and calcium. The elevation of calcium triggers calmodulin-dependent kinase (CaMK) enzymes. CaMKII, forming 1–2% of brain protein, supports neurotransmitter release, glycogen use, and synaptic plasticity, representing the brain’s ability to strengthen or weaken neural connections regarding learning and memory [5]. CREB signaling may participate in the development of epilepsy and offers a potential target for creating first-line antiepileptic drugs. It could also act as a key indicator for epilepsy screening, diagnosis, and outcome prediction [6].

Lacosamide, a newer generation antiepileptic drug, approved in 2008 by the Food and Drug Administration (FDA), offers good efficacy, advantageous bioavailability, minimal drug-to-drug interactions and good tolerability [7]. Common side effects include headaches, nausea, dizziness, and diplopia [8]. It can be used in the management and treatment of partial-onset seizures or as an adjunctive in primary generalized tonic–clonic seizures [9]. Lacosamide’s mechanism of action is based on slow inactivation voltage-gated sodium channels enhancement as well as inhibition of the collapsin response mediator protein 2 (CRMP-2) [2,10]. Lacosamide has shown efficacy in several rodent models of epilepsy in addition to potential potency against neuropathic inflammatory pain [11].

Repetitive transcranial magnetic stimulation (rTMS) is a non-invasive method of cortical stimulation that can affect synaptic plasticity, generating enduring effects in the brain. This method of brain stimulation may either be excitatory or inhibitory based on the application frequency. Lower frequencies of less than 1 Hz have an inhibitory effect, thus making neurons less likely to fire an action potential, while higher frequencies (>5 Hz) have an excitatory effect, making neurons more likely to fire an action potential [12]. Based on its characteristics, rTMS can be used in several health conditions and diseases such as depression, post-operative pain, Alzheimer’s disease, schizophrenia, multiple sclerosis (MS), and epilepsy [13]. There is also some evidence that rTMS could be successfully used in psychiatric disorders like generalized anxiety disorder (GAD), bipolar disorder, and in post-traumatic stress disorder (PTSD) [14].

Numerous clinical studies have explored the use of rTMS and it has also been used for all those conditions and diseases mentioned above since the early 1990s [15]. Regardless of its extensive use and the potential benefit, in vitro and in vivo studies remain limited. Moreover, there are very few studies combining the use of rTMS with market-approved drugs for any condition. Here, we aim to elucidate the potential benefit in the combination of rTMS and the antiepileptic drug lacosamide when used on an in vitro model of neuronal excitability. In our experiment, we used the human neuroblastoma cell line SH-SY5Y, after implementing a differentiation protocol based on retinoic acid and brain-derived neurotrophic factor (BDNF), in order to achieve cultured mature neurons, both in morphology and physiology, that have lengthened neurites and express mature neuronal markers [16,17]. To imitate neuronal excitability in vitro, we used potassium chloride, which leads to elevated intracellular calcium ion concentration, a common characteristic observed during the epileptic state [18,19].

The SH-SY5Y cell line may differentiate into neuron-like cells with distinct phenotypic characteristics including the cholinergic, dopaminergic, or adrenergic phenotype, dependent upon the culture conditions. The use of retinoic acid to promote differentiation has been linked to either the cholinergic or dopaminergic phenotype with the relevant markers [20]. Cholinergic markers include the enzyme choline acetyltransferase (ChAT) which is responsible for the synthesis of acetylcholine (ACh) and acetylcholinesterase (AChE), the enzyme responsible for breaking down acetylcholine which are both upregulated during differentiation [21]. The addition of the growth factor BDNF in the differentiation protocol also significantly enhances neurite density and resembles the characteristics of cholinergic neurons regarding both their enzymatic activities and the cholinergic markers [21]. While the SH-SY5Y cell line is primarily used in neurotoxicity and neuroinflammation studies, in its differentiated state, it is also characterized by neuron-like excitability which supports its use for investigating KCl-induced neuronal hyperexcitability [22,23].

In the present in vitro study, we examined the effects of magnetic stimulation (rTMS) and lacosamide, when used individually or combined, and their impact on intracellular calcium ion concentration utilizing the fluorescent chemical Ca^2+^ indicator, Fluo-4, AM. The combination of lacosamide and TMS in vitro represents a novel approach to studying antiepileptic therapies, as prior studies have primarily examined antiepileptic drugs or rTMS independently. Our study is among the first to explore their combined effects on SH-SY5Y cells, offering a controlled foundation to explore cellular mechanisms underlying their antiepileptic potential.

## 2. Materials and Methods

### 2.1. Cell Culture

The SH-SY5Y human neuroblastoma cell line was purchased from Sigma-Aldrich (St. Louis, MO, USA). Cell cultures were maintained in controlled conditions in an incubator at 37 °C and 5% CO_2_ and were passaged continuously for the need of the experiments. Cells were grown in T25 cell culture flasks in EMEM supplemented with glutamine, penicillin/streptomycin, and fetal bovine serum (FBS), then transferred to Petri dishes before the initiation of the differentiation protocol for each experiment. For our experiment, cells were categorized into four groups, namely control, rTMS, lacosamide and rTMS, and lacosamide.

### 2.2. Chemicals

Dulbecco’s Modified Eagle’s Medium (DMEM), Eagle’s Minimum Essential Medium (EMEM), Pluronic F-127 and supplement to basal growth medium, Fetal bovine serum (FBS), were purchased from Sigma-Aldrich. The differentiation medium Neurobasal medium without phenol red was purchased from Gibco™. Imaging BrainPhys™ Neuronal Medium was purchased from Stemcell Technologies, Vancouver, BC, Canada. Penicillin/Streptomycin (Pen/Strep), N2 supplement, Trypsin-EDTA (1×), and Dimethylsulfoxide (DMSO) were purchased from Gibco™, Thermo Fisher Scientific, Waltham, MA, USA. Human brain-derived neurotrophic factor (BDNF) was purchased from Abbkine (Abbkine Scientific Co., Ltd., Wuhan, China). Glutamine (100×) 200 mM was acquired from Biowest, Nuaillé, France, and Fluo-4, AM (acetoxymethyl ester) from Invitrogen (by Thermo Fisher Scientific, Waltham, MA, USA). Lacosamide was used as a solution for injection at 10 mg/mL, (Vimpat, UCB Pharma S.A., Brussels, Belgium). A solution of potassium chloride (KCl, 50 mM) was utilized to provoke neuronal excitation and elevate the intracellular calcium concentration.

### 2.3. Differentiation Protocol

**Figure 1:**
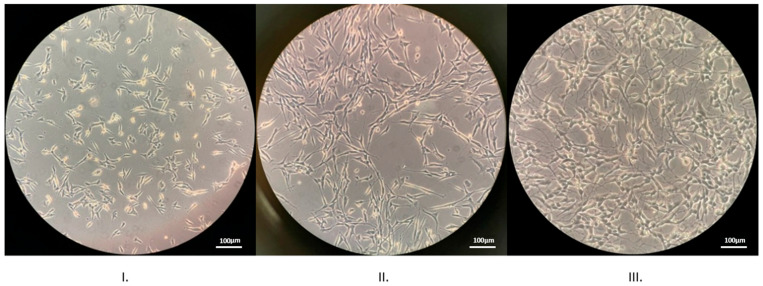
Representative photos showing the morphology of SH-SY5Y cells during differentiation. (**I**). Cells before initiating the differentiation protocol, (**II**). Day 3, end of phase 1 of the differentiation protocol, (**III**). Day 6, end of phase 2 of the differentiation protocol. Over 99% of differentiated cells exhibited neuritic connections with neighboring cells.

The differentiation protocol used was the 6-day protocol, advanced by Forster et al., comprising two separate phases with different cell culture media between them [17]. Cells were harvested and centrifuged for 10 min at 1500 rpm before they were transferred to Petri dishes (35 mm × 10 mm) where they remained for a period of 24 h in the incubator before the initiation of the differentiation protocol. The phase 1 medium consisted of DMEM with high glucose (25 mM) minus sodium pyruvate, penicillin/streptomycin (1%), L-glutamine (4 mM), and all-trans retinoic acid (10 µM). After 72 h, phase 1 medium was replaced with phase 2 differentiation medium consisting of Neurobasal medium (NB) without phenol red, brain-derived neurotrophic factor (BDNF) (50 ng/mL (*v*/*v*)), 100× N-2 supplement (1%), L-glutamine (1%), and Penicillin–Streptomycin (1%). After 72 h, 6 days after the beginning of the differentiation protocol (Figure 1), cells were prepared for loading with the fluorescent dye Fluo-4, AM, and calcium imaging.

### 2.4. Calcium Imaging

For intracellular calcium quantification, the fluorescent dye Fluo-4, AM was used. This fluorescent dye has the capacity to produce augmented fluorescence when it binds to calcium ions inside loaded cells. For the needs of our protocol Fluo-4, AM was solubilized in DMSO at a concentration of 50 μg /0.2 mL DMSO. In each cell culture, the differentiation phase 2 medium was replaced by the loading buffer containing the fluorescent dye (5 μM), in addition to Pluronic F-127 (6 μL) diluted in DMEM for a final loading medium of 1.5 mL. Cells were then placed in the incubator. Thirty minutes later, cells were left resting at room temperature, for an additional 15 to 30 min, right after the loading buffer was replaced by DMEM with 15% FBS, in the absence of light. Cells were then ready for calcium imaging. For each cell culture, we performed one recording before and one identical recording immediately after adding potassium chloride. The neuronal medium used throughout the recordings was BrainPhys, an optimized medium, with a composition similar to the extracellular environment of the central nervous system (CNS) extracellular environment, that facilitates the activity and basic synaptic functions of neurons [24]. Our protocol included 10 trials of 5120 ms (acquisition duration), or 512 frames with 10 ms frame interval, and 10 s intervals between the trials for every cell culture. In each microscope field, 10 traces each one in a different cell were chosen with a kernel size of 2 pixels to measure the corresponding fluorescence values. After the addition of potassium chloride and the second identical imaging protocol performed, the exact same traces were contrasted to measure and quantify the fluorescence fluctuation. To normalize fluorescence values and avoid the confounding results of non-uniform loading delta F over F was used (%ΔF/F), or the final fluorescence value minus the initial fluorescence value divided by the resting intensity value (fluorescence prior to the use of potassium chloride) [25,26]. In our study, we used a Carl Zeiss Axio ExaminerZ1(Carl Zeiss Microscopy GmbH, Jena, Thuringia, Germany) microscope with a water immersion objective lens (Zeiss 10×/0.3). Optical recordings were performed using a high- speed CMOS camera (NeuroCMOS-SM128, Redshirt Imaging Inc., Decatur, Georgia) which was attached to the C-mount port of an upright epifluorescence microscope. Data were obtained by Neuroplex version 9.9.8. and analyzed to compare and quantify fluorescence values between different groups.

### 2.5. Lacosamide and rTMS

**Figure 2:**
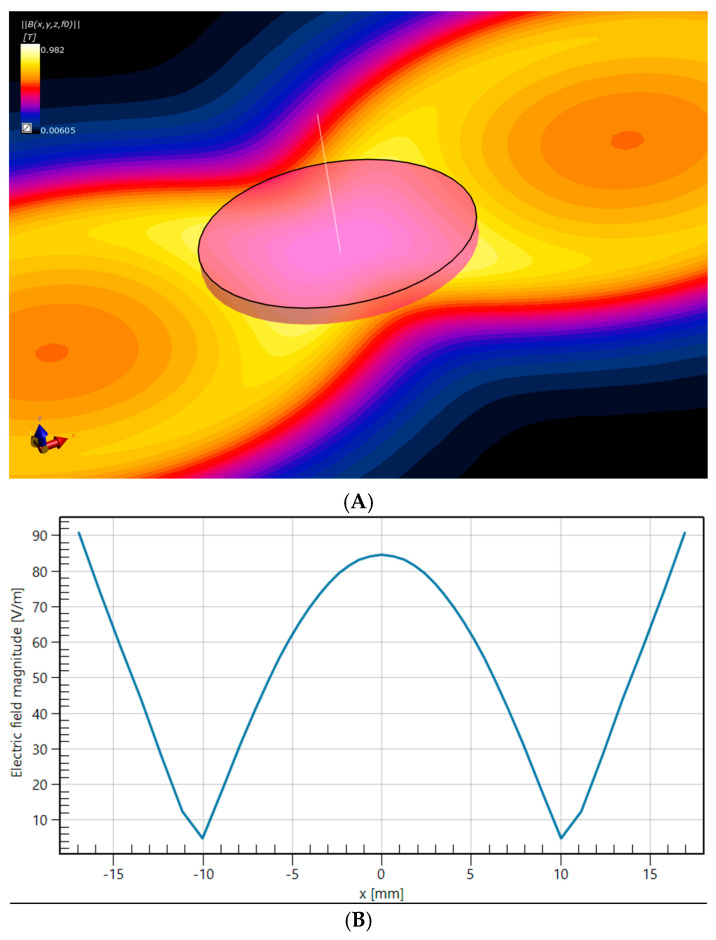
(**A**). Magnetic field distribution generated by the figure-8 coil in the plane lying at the bottom of the Petri dish. (**B**). Electric field distribution along the diameter of the Petri dish (at the same plane) which is parallel to the large axis of the figure-8 coil.

Cells were treated with lacosamide (at a concentration of either 30 or 300 μM) 30 min prior to the Calcium imaging. The high-performance TMS stimulator, MagPro R30 MagVenture paired with a figure-8 coil (model C-B60), was used for magnetic stimulation of the cell cultures. We implemented two trains of 100 pulses each, at 100% amplitude in the low frequency of 1 Hz. To ensure the maximum temperature of the coil never exceeded 40 °C during magnetic stimulation, we monitored it using an infrared thermometer. The short duration of the stimulation ensured that cell cultures remained at a temperature below 37 °C during the whole experiment. The entire magnetic stimulation protocol took place in the dark to avoid the effects of light due to the photosensitive nature of the calcium indicator. To accomplish the greatest magnetic intensity, cell culture dishes were placed on top of the coil at the conceivable point of intersection both horizontally and vertically. The induced electric field in the Petri dish was determined using Sim4Life (version 7.2, ZMT Zurich MedTech AG, Zürich, Switzerland). The electrical conductivity of the medium was configured to 1.5 S/m, as reported in the study by Khire et al. [27]. A harmonic current with a frequency of 4.5 kHz was used, matching the basal frequency of a 222 μs rectangular pulse. The peak magnetic field strength achieved at the base of the culture medium was 0.98 T (Figure 2A), while the maximum electric field, induced within the culture medium, reached 90 V/m (Figure 2B) [28].

### 2.6. Statistical Analysis

The statistical analysis included standard descriptive statistics to assess the %ΔF/F within the four different categories of treatment (control, TMS, lacosamide, and TMS and lacosamide). Data distributions were visualized using box plots. The non-parametric Kruskal–Wallis test was used to evaluate the distributional differences in %ΔF/F among the four groups. Dunn’s test was applied for multiple pairwise comparisons in the post hoc analysis, paired with the Benjamini–Hochberg method to adjust for multiple comparisons [29]. A statistical significance threshold of 0.05 was set. Analysis was carried out using SPSS software (version 22.0) and the R programming language (version 4.4.1).

## 3. Results

An elevation of intracellular calcium demonstrated by a corresponding elevation of fluorescence intensity, following the application of KCl (50 mM), was consistently observed throughout our experiments. These observations have also been reported in the literature by many researchers [30,31].

We aimed to elucidate if this fluorescence augmentation is mitigated when a pharmacological intervention with an antiepileptic drug, a magnetic stimulation intervention, or their combination is applied to the cell cultures prior to the addition of potassium chloride in the imaging medium. For the normalization of the fluorescence values observed, delta F over F (%ΔF/F) was used in all experiments. To apply delta F over F, the initial fluorescence value was deducted from the final fluorescence value, and their subtraction result was divided by the resting fluorescence intensity. All data are presented in Table 1, representing the mean, the median percentage change in fluorescence (%ΔF/F), and the standard deviation, which quantifies intracellular calcium elevation in SH-SY5Y cell cultures following the addition of KCl. The %ΔF/F reflects the relative increase in intracellular calcium levels compared to baseline fluorescence. Lacosamide was applied on cell cultures at either 30 μΜ, an achievable plasma concentration in clinical practice, or at the 10-fold concentration of 300 μM. Pilot experiments with the lower concentration (30 μΜ) did not show any statistically significant results. All experiments, as described in Table 1, have been performed at a concentration of 300 μM [32,33].

We found an increase in fluorescence levels in cell cultures after the application of potassium chloride on the culture medium. The highest increase was observed in the control groups, where no pharmacological or magnetic stimulation was applied. The lowest fluorescence increase was detected in cell cultures where both lacosamide and magnetic stimulation were applied. The %ΔF/F exhibited a median value of 66.70 in the control group, 23.70 in the TMS group, 17.11 in the lacosamide group, and 9.15 in the TMS and lacosamide group (Table 1).

**Figure 3:**
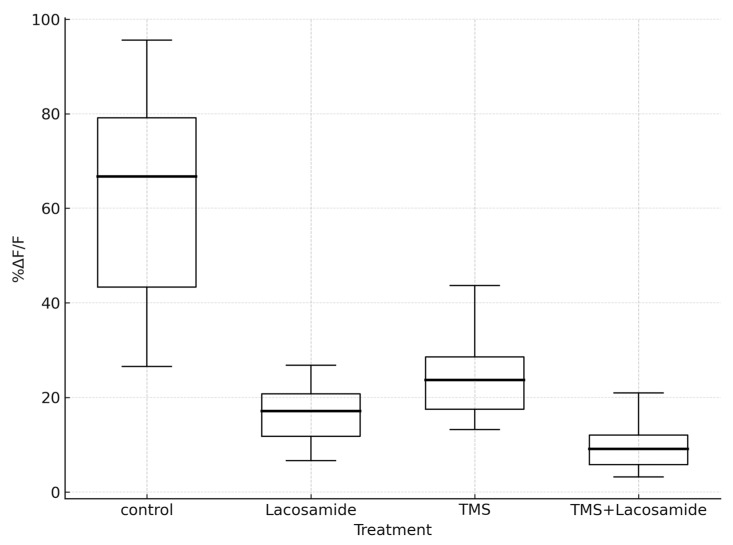
Box plots describing the percentage change in fluorescence in control, lacosamide, TMS, and TMS and lacosamide groups.

**Figure 4:**
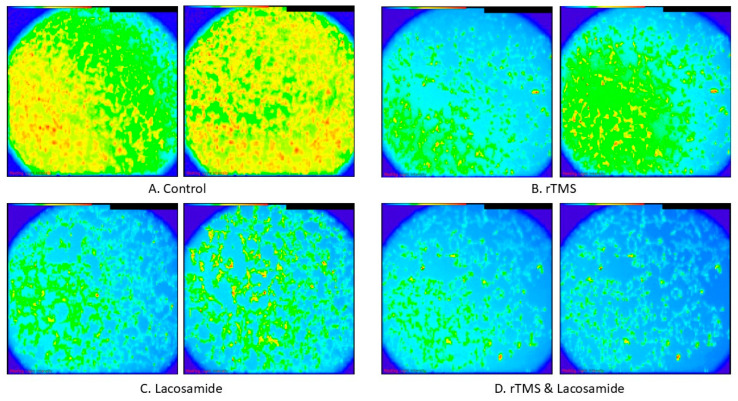
Representative images of intracellular Ca^2+^ in various SH-SY5Y culture groups, using the fluorescent calcium indicator Fluo-4 AM, displaying fluorescence intensity with blue indicating low Ca^2+^ levels and red high Ca^2+^ levels. (**A**). Control cultures before and after the addition of KCl, (**B**). rTMS cells cultures before and after KCl addition, (**C**). lacosamide cell cultures before and after the addition of KCl, (**D**). rTMS and lacosamide cell cultures before and after the addition of KCl.

The distribution characteristics of the %ΔF/F for the four different treatments are shown in Figure 3. The Kruskal–Wallis test showed that the variation in the distributions of %ΔF/F within these four groups was statistically significant (*p*-value < 0.001). When the pairwise differences were assessed in the post hoc analysis, it was found that the control group, which exhibited the highest median value (66.70), differed statistically significantly compared to all the remaining three groups (TMS, lacosamide, and TMS and lacosamide) with adjusted *p*-values < 0.001, <0.001, and <0.001, respectively (Table 1). Moreover, the TMS and lacosamide group, which exhibited the lowest median value (9.15), exhibited a statistically significant difference when compared to both the TMS group (adjusted *p*-value < 0.001) and the lacosamide group (adjusted *p*-value = 0.003) (Table 1). TMS and lacosamide also exhibited a statistically significant difference in distribution (adjusted *p*-value = 0.018). Calcium imaging photos are shown in Figure 4.

## 4. Discussion

In the present study, we investigated the intracellular calcium concentration variations under different conditions, specifically the impact of rTMS, the use of the antiepileptic drug lacosamide, and their combination in a human-derived neuronal cell line. For this experiment, the SH-SY5Y cell line was differentiated into a mature neuron-like cell line. During our pilot studies, we accomplished two distinct differentiation protocols. First, we performed the 17-day protocol by Shipley et al. using retinoic acid, extracellular matrix proteins, and certain neurotrophic factors [34]. Subsequently, we performed the shorter in duration differentiation protocol by Forster et al., the 6-day, two-step process, based on retinoic acid, BDNF and Neurobasal, a medium formulated to appraise the neuronal cell requirements. We ultimately utilized this protocol throughout all our main experiments as it was more time-efficient and required less laboratory resources while the final confluency at the end of the differentiation protocol was more advantageous [17]. The SH-SY5Y cell differentiation process, adapted from Encinas et al., has been optimized for efficiency using N2-supplemented Neurobasal medium in the second phase of differentiation [35]. While undifferentiated SH-SY5Y cells form clumps after the initiation of the differentiation protocol, by the end of the first phase, or day 3, these clumps are reduced, and cells begin to extend neurites. By day 6, when the differentiation protocol has been completed, the cells are evenly spread and are connected through a network of branched neurites. Although only 2% of undifferentiated cells have neurites, 99% of cells display neurites once fully differentiated state [17].

In in vitro models of seizure liability, there are certain methods that can be implemented to induce epileptiform activity, namely the manipulation of ion levels and pharmacological induction. In the former category, elevating potassium, or reducing extracellular calcium or magnesium, may exert epileptiform activity on neurons. The latter category includes the use of certain drugs like 4-aminopyridine (4-AP) or GABA antagonists. Measuring the intracellular calcium concentration through a period of time with calcium imaging constitutes a common method to explore general neuronal activity and calcium oscillations [18]. Among all these methods of seizure liability, we chose to elevate potassium levels with the addition of potassium chloride to the bathing solution.

Lacosamide’s main antiepileptic mechanism of action is based on slow inactivation of voltage-gated sodium channels which are endogenously expressed in neuroblastoma cell line SH-SY5Y [10,36]. Magnetic stimulation has an impact on α-amino-3-hydroxy-5-methyl-4-isoxazolepropionic acid receptor (AMPA) and N-methyl-D-aspartate (NMDA) ionotropic receptors which are also expressed on our cell line [37,38]. Gap junctions, formed by connexin proteins, enable the exchange of ions and small molecules between cells, promoting synchronized neuronal firing associated with epilepsy. In SH-SY5Y neuroblastoma cells, which express connexin 43, gap junctions likely enhance calcium wave propagation during epileptiform activity induced by potassium chloride, contributing to increased calcium levels [39,40]. Recent studies have suggested that lacosamide’s activity may be partially attributed to its ability to reduce functional coupling through gap junctions, which may contribute to the results observed in the present study [41]. Limited literature exists on the effects of TMS on gap junctions. A study demonstrated that rTMS reduces connexin 43 expression in astrocytes in mice, enhancing motor recovery, which activates the mTOR pathway and modulates autophagy, suggesting potential implications for TMS in regulating gap junction function [42]. In the field of epilepsy, TMS modulates molecular mechanisms by activating NMDA receptors, inducing cortical neuronal depolarization, leading to dendrite remodeling and promoting long-term potentiation. It can also modulate GABAergic neurons, activating antiapoptotic pathways facilitating neuroprotection and triggering actin cytoskeleton rearrangements affecting synaptic function [43,44].

For the needs of our study, we tested lacosamide in two concentrations, 30 μM and 300 μM. Neither of them caused any alterations in confluency or viability of the cells but the highest dose was the most potent as it is also highlighted in the literature. No statistically significant results have been extracted at the lower concentration (30μM) and all experiments, as described in Table 1, have been performed at a concentration of 300μM [31,41]. The higher dose was chosen to enhance the sensitivity of our in vitro model for detecting effects as it consistently produced strong responses in a controlled setting while this does not represent therapeutic dosing levels [32,33]. In a study related to epilepsy, the researchers examined the efficacy of lacosamide both in vitro, in neocortical brain slices, and in vivo, on C57BL/6J mice, and found that it is dose-dependently effective without augmenting apoptosis in either one of them. A reduction in epileptiform activity was observed in vitro and a decrease in seizures’ frequency in vivo [45]. The effectiveness of lacosamide was also recorded in vitro by a study on multiple antiepileptic drugs and antidepressants on rat hippocampal slices. Lacosamide reduced the duration of seizure-like events (SLEs) and the interictal period duration through full repression of epileptiform activity [46]. Another in vitro study used three new antiepileptic drugs, namely levetiracetam, zonisamide, and lacosamide, in an acute seizure model provoked by 4-aminopyridine (4-AP). They found that lacosamide at high concentrations fully abolished seizure-like events (SLEs) while they also highlighted the significance and contribution of in vitro models in the field of epilepsy [47]. These results are in agreement with our study, where cell cultures treated with lacosamide showed statistically significant reduced fluorescence augmentation upon the addition of potassium chloride in comparison to the control groups with a %ΔF/F median value of 17.11 versus 66.70, respectively (adjusted *p*-value < 0.001).

It has been described in the literature that low frequency rTMS, with a frequency below 1 Hz, is associated with reduced motor evoked potentials, alleviated cortical excitability, and long-term depression (LTD) [48]. In contrast, high frequency rTMS, with a frequency of 5 Hz or more, is described as excitatory, leading to long-term potentiation and elevated synaptic strength [12].

Studies have implemented the use of magnetic stimulation in the cure of epilepsy. One study used 4-Aminopyridine to induce ictal activity on rat brain slices before applying repetitive low-frequency magnetic stimulation, which resulted in inhibitory outcomes with the optimal effectiveness observed at 1Hz, concluding that the findings may be beneficial in the management of pharmacoresistant epilepsy [49]. Regarding clinical research, a study performed on 25 healthy individuals, also using low-frequency magnetic stimulation (1 Hz) in combination with a 6 Hz priming stimulation, found more robust depression and long-term depression (LTD) which did not deteriorate 1 h following its application [50]. A double-blind study aimed to elucidate the antiepileptic effect of levetiracetam on humans and the existence of any resemblance to in vivo outcomes. The researchers recruited ten healthy volunteers and found that levetiracetam (2000 mg) resulted in a notable elevation of motor threshold with reduced motor evoked potential (MEP) levels, findings that were in agreement with the in vivo ones. The anticonvulsive effects of levetiracetam were invigorated by the implementation of TMS, where the antiepileptic drug abolished the activation of corticospinal neurons, effectuated by TMS [51]. Another study on healthy individuals investigated the effects of two antiepileptic drugs, namely lacosamide and carbamazepine, in combination with magnetic stimulation (TMS). They found that both drugs considerably elevate the motor threshold of TMS compared to the placebo, with lacosamide having a dose-depended potency in reducing neuronal membrane excitability levels [52]. These experiments also support our findings where cell cultures treated with low-frequency magnetic stimulation appeared to showcase decreased fluorescence values compared to our control groups after treatment with potassium chloride with %ΔF/F median values of 23.70 (TMS) compared to 66.70 (control) (adjusted *p*-value < 0.001).

To our knowledge, there are no in vitro studies in the field of epilepsy or epileptiform activity examining the use of lacosamide in combination with magnetic stimulation. The findings of the present study suggest that cell cultures where low frequency rTMS has been applied in combination with lacosamide showed the lowest fluorescence increase among all cell cultures. According to our post hoc analysis, the TMS and lacosamide group showed a statistically significant difference when compared to both the TMS group and the lacosamide group, while a statistically significant difference was observed with lacosamide, showing a more potent effect than TMS alone (%ΔF/F median values of 17.11 for lacosamide compared to 23.70 for TMS (adjusted *p*-value 0.018)).

We recognize certain limitations in the present study. While special care and attention have been taken to avoid variations in environmental factors such as temperature and lighting, small alterations may have occurred. One limitation of this study is the limited number of replicates, which may reduce statistical power to detect slight differences, while future studies with larger sample sizes will improve statistical power. Moreover, we performed our experiments in a drug concentration based on its ability to elicit strong responses under controlled in vitro conditions without reflecting therapeutic dosing levels. We acknowledge that further in vivo studies are essential in order to establish the minimum effective dose and ensure clinical translation. Even though we used human derived mature neurons, in vitro neuronal cultures, in general, may not mimic the complex three-dimensional architecture, synaptic networks, and interactions observed when various cell types found in the human brain, such as astrocytes and microglia, are present, making our approach a simplification of the complex pathophysiological characteristics of epilepsy, limiting the direct translation to in vivo seizure activity. Moreover, electrophysiological recordings were not included in our study. This study relied on calcium imaging to assess neuronal excitability, without complementary electrophysiological recordings such as patch clamps. These techniques could provide direct measures, enhancing mechanistic insights. Future studies incorporating electrophysiological approaches will strengthen our understanding of lacosamide–rTMS effects. Furthermore, the rTMS protocol in this study used a 1 Hz frequency without calibration to motor thresholds or phosphene presence, as typically performed in clinical settings limiting the translatability of our findings to human applications. The SH-SY5Y cell culture model cannot replicate the complex neuronal networks, excitability thresholds, or electromagnetic dynamics of the human brain, which are critical for therapeutic rTMS efficacy. To enhance the clinical relevance of our findings and facilitate the translation of in vitro results to in vivo applications, several limitations have to be addressed. Future studies should incorporate mixed cultures or organoid models structured in a three-dimensional environment that will better resemble the structure and function of the human brain and assess their effects on treatment results. Cell culture diversity may have a vital role in modulating brain activity, including neurotransmitter regulation and calcium dynamics, which may significantly in-fluence the effects of rTMS and pharmacological intervention, such as the antiepileptic drug lacosamide. Several steps have to be taken in order to translate our in vitro findings into clinical practice. In vivo studies, including animal models of epilepsy, are needed to confirm the reduction in neuronal excitability observed in our study and further elucidate the underlying mechanisms. Clinical trials should then assess the optimization of rTMS parameters, regarding frequency and intensity, as also the optimal lacosamide dosage in drug-resistant epilepsy patients. The drug concentrations of lacosamide used in this study were selected based on their ability to elicit robust responses under controlled in vitro conditions and do not reflect therapeutic dosing levels used in clinical practice, where complex pharmacokinetic and pharmacodynamic factors influence lacosamide’s effects in humans, namely systemic metabolism, blood–brain barrier penetration, and interactions with diverse neuronal cells. This limits the direct translatability of our findings to clinical settings, as the observed effects on intracellular calcium levels may not fully represent lacosamide’s antiepileptic actions in vivo. Our study should therefore be viewed as a simplified mechanistic investigation of these effects rather than as evidence of direct clinical efficacy in humans. Ultimately, they could guide the development of dosing guidelines and rTMS stimulation parameters in order to achieve efficacy and maximize therapeutic synergy.

## 5. Conclusions

Our findings demonstrate that both lacosamide and rTMS reduce KCl-induced calcium influx in differentiated SH-SY5Y neuroblastoma cells, with their combination showing the greatest effectiveness, offering a novel in vitro framework for studying combination therapies. These results suggest that combining rTMS with an antiepileptic drug may represent a promising strategy for addressing drug-resistant epilepsy. Future studies should investigate long-term effects and incorporate electrophysiological recordings to further validate these findings before progressing to in vivo models of epilepsy and, ultimately, clinical trials and clinical applications.

## Figures and Tables

**Table 1:** (A) Descriptive statistics are displayed for the four groups (control, TMS, lacosamide, and TMS and lacosamide), corresponding to three replicates, which each replicate consisting of 10 traces per treatment. Median, mean %ΔF/F values are presented with standard deviation values to indicate variability. (B) Post hoc analysis is displayed, corresponding to the Kruskal–Wallis test, which showed that the distributions of %ΔF/F within these four groups was overall statistically significant (*p*-value < 0.001). Post hoc pairwise comparisons were determined by Dunn’s test. The values of Dunn’s test statistic, the corresponding unadjusted *p*-values and the adjusted *p*-values, after employing the Benjamini–Hochberg procedure to account for multiple comparisons, are displayed in separate columns.

(**A**)			
**Groups**	**Median**	**Mean**	**Std. Deviation**
Control	66.70	62.88	22.86
TMS	23.70	23.12	7.27
Lacosamide	17.11	16.82	5.81
TMS + Lacosamide	9.15	9.84	4.73
(**B**)			
**Group 1/Group 2**	**Dunn’s Test Statistic**	**Unadjusted** ***p*-Value**	**Adjusted** ***p*-Value (Using Benjamini–Hochberg)**
Control vs. TMS	32.47	<0.001	<0.001
Control vs. Lacosamide	51.89	<0.001	<0.001
Control vs. TMS + Lacosamide	77.17	<0.001	<0.001
TMS vs. Lacosamide	19.42	0.018	0.018
TMS vs. TMS + Lacosamide	44.70	<0.001	<0.001
Lacosamide vs. TMS + Lacosamide	25.28	0.002	0.003

## Data Availability

All the data supporting the findings of this study are available from the corresponding author upon reasonable request.

## Abbreviations

The following abbreviations are used in this manuscript:

rTMS	repetitive transcranial magnetic stimulation
DRE	Drug resistant epilepsy
FDA	Food and drug administration
MRI	Magnetic resonance imaging
ACh	Acetycholine
AChE	Acetylcholinesterase
ChAT	Choline acetyltransferase
CaMK	Calmodulin-dependent kinase
CREB	cAMP response element binding
CRMP-2	Collapsin response mediator protein 2
MS	Multiple sclerosis
GAD	Generalized anxiety disorder
PTSD	Post-traumatic stress disorder
BDNF	Brain-derived neurotrophic factor
DMEM	Dulbecco Modified Eagle’s Medium
EMEM	Eagle’s Minimum Essential Medium
DMSO	Dimethylsufloxide
FBS	Fetal bovine serum
CNS	Central nervous system
4-AP	4-aminopyridine
SLEs	Seizure-like events
LTD	Long term depression
MEP	Motor evoked potential
PD	Pharmacodynamic
PK	Pharmacokinetic
